# Four-week IgE/baseline IgE ratio combined with tryptase predicts clinical outcome in omalizumab-treated children with moderate-to-severe asthma

**DOI:** 10.1515/med-2025-1176

**Published:** 2025-05-21

**Authors:** Min Lu, Jing Zhao, Mei Zeng, AiMin Zhu, JinFen Li

**Affiliations:** Department of Pediatrics, Suzhou Research Center of Medical School, Suzhou Hospital, Affliated Hospital of Medical School, Nanjing University, Suzhou, Jiangsu, 215153, China; Department of Child Health Care, Ezhou Central Hospital, Ezhou, Hubei, 436000, China; Department of Emergency Medicine, The General Hospital of Western Theater Command PLA, Chengdu, Sichuan, 610000, China; Department of Pediatrics, The 940th Hospital of Joint Logistic Support Force of Chinese People’s Liberation Army, Lanzhou, Gansu, 730050, China; Department of Pediatrics, The Fourth Affiliated Hospital of Soochow University, No.9, Chongwen Road, Suzhou Industrial Park, Suzhou, Jiangsu, 215000, China

**Keywords:** serum IgE, tryptase, omalizumab, asthma, efficacy

## Abstract

**Objective:**

The aim of this study was to investigate the feasibility of the 4-week IgE/baseline IgE ratio and tryptase to predict the clinical efficacy of omalizumab in the treatment of pediatric moderate-to-severe asthma.

**Methods:**

Moderate-to-severe asthma patients were selected, and their baseline IgE levels were recorded, and the IgE levels were tested again after 4 weeks of treatment with omalizumab to calculate the 4-week IgE/baseline IgE ratio. Serum tryptase was measured. Treatment efficacy was assessed. Patients were divided into response and non-response groups. Receiver operating characteristic curves were plotted for the sensitivity and specificity of the indices in predicting response to omalizumab treatment.

**Results:**

Serum total IgE levels increased to 655.89 IU/mL, serum tryptase was 5.31 ng/mL after 4 weeks of treatment, which was higher than at baseline. There was a significant difference in total serum IgE between children in response and non-response groups after 4 weeks of treatment. The response group children had a higher 4-week IgE/baseline IgE ratio, whereas tryptase was lower. Combined metrics had moderate predictive value for the efficacy of omalizumab therapy.

**Conclusion:**

The 4-week IgE/baseline IgE ratio combined with tryptase can predict, to a certain extent, the clinical efficacy of omalizumab in the treatment of pediatric moderate-to-severe asthma.

## 1 Introduction

Asthma is a frequent chronic respiratory disease among children, and its global prevalence has been gradually increasing [1]. Asthma is a heterogeneous disease with a complex pathogenesis and multiple risk factors, and so far it is still incurable and can only be controlled [2,3]. Since 2006, the Global Initiative for Asthma has emphasized two core concepts: “asthma control” and “comprehensive asthma management,” both centered on achieving long-term symptom control and reducing risks [4]. The situation of asthma control is still grim [5], primarily because of children experiencing moderate-to-severe asthma [6]. Therefore, there is an urgent need to improve the prevalence and care of children with moderate-to-severe asthma.

IgE is a vital element in treating allergic conditions and is a primary contributor to airway inflammation in cases of allergic asthma [7]. Therefore, targeted therapy against IgE may help alleviate clinical symptoms in children with asthma. Omalizumab, a monoclonal antibody specifically designed for IgE, is the first targeted drug approved worldwide for the treatment of moderate-to-severe asthma [8]. Omalizumab specifically binds free IgE and blocks the allergic cascade by preventing IgE from binding to high-affinity FcεRI receptors on mast cells, antigen-presenting cells, and other inflammatory cells [9]. Therefore, anti-IgE therapies are effective in alleviating clinical symptoms in children, minimizing the number of acute asthma flare-ups, reducing the need for oral medications, and enhancing quality of life, asthma symptoms, eosinophilic inflammation, and airway remodeling, as well as have a significant effect on asthma-associated allergic diseases [10,11]. It was only on March 18, 2018 that the first batch of omalizumab in China was qualified and formally applied to children with moderate-to-severe allergic asthma ≥6 years old [12]. Omalizumab elicited a therapeutic response in 91.5% of patients following 16 weeks of treatment [13]. However, based on limited clinical experience and data, the clinical effectiveness and cost-effectiveness of omalizumab for the treatment of severe persistent asthma in children aged 6–11 years still warrants further investigation.

The effectiveness of omalizumab is closely linked to the presence of free IgE and omalizumab–IgE complexes following treatment. Research has shown that the levels of free IgE and omalizumab in the serum of patients treated with omalizumab do not determine clinical success and do not impact the decision to either continue or halt treatment [14]. Omalizumab–IgE immune complexes interfere with the usual measurement techniques for total serum IgE, preventing the distinction between free IgE and these complexes. Omalizumab–IgE complexes are excreted more slowly relative to free IgE, and after anti-IgE therapy, most patients will exhibit a rapid and significant increase in serum total IgE levels from baseline values over a period of time [15]. Currently, there appears to be no relevant studies confirming the excretion time of omalizumab–IgE complexes. However, pharmacokinetic studies have shown that following subcutaneous administration of omalizumab, drug serum levels reach a maximum at day 7–8 [16]. The clearance half-life of omalizumab is estimated to be approximately 24–26 days (24 days in serum in chronic idiopathic urticaria and 26 days in asthma data) [17]. Furthermore, a 4-week IgE/baseline IgE ratio is noted as a biomarker for a favorable response to omalizumab in cases of chronic spontaneous urticaria [18].

Typically, tryptase degranulation is connected with immune response development, allergic reactions, inflammation, and tissue remodeling [19]. Tryptase is primarily produced and released in significant amounts by mast cells, with basophils releasing smaller quantities. Upon activation, mast cells release tryptases, which can affect the contraction and relaxation of airway smooth muscle, worsening airway inflammation and hyperresponsiveness [20].

While omalizumab is effective in most moderate-to-severe asthma cases, there is an absence of precise clinical indicators of its effectiveness. Therefore, the aim of this study was to investigate the potential value of serum IgE and tryptase levels in predicting the efficacy of omalizumab in the treatment of pediatric severe asthma.

## 2 Materials and methods

### 2.1 Patients

A total of 68 children with moderate-to-severe allergic asthma (age ≥6 years) treated with omalizumab admitted at The Affiliated Suzhou Hospital of Nanjing University Medical School from October 2022 to June 2024 were collected. Inclusion criteria: (1) Patients ≥6 years of age and <18 years of age; (2) patients with moderate-to-severe asthma according to the diagnostic criteria of Guidelines for bronchial asthma prevent and management (2020); moderate asthma was defined as fully controlled by level 3 asthma medication, and severe asthma was defined as fully controlled or not fully controlled by level 4 or level 5 treatment; (3) serum total IgE ≥ 35 IU/mL or positive results of skin prick test; (4) patients whose baseline weights were all within the omalizumab dose schedule. Exclusion criteria: (1) patients receiving strain-specific immunotherapy or other targeted therapies during the treatment period; (2) those with incomplete clinical data and interruptions in treatment or follow-up; (3) elevated levels of IgE and medium-chain triglycerides due to reasons other than anaphylaxis (e.g., parasitic infections, high-IgE syndrome, fungal infections, etc.); (4) the guardians who did not sign the written informed consent form. Forty-three patients were finally included.

### 2.2 Clinical data

Baseline data of patients prior to the administration of omalizumab were obtained in the electronic medical record. Demographics included age, gender, height, and weight. Clinical characteristics included duration of asthma, comorbid allergic diseases, asthma severity, basic asthma treatment regimen, inhaled corticosteroids (ICS)/long-acting β_2_-adrenoceptor agonists (LABA) dosage, predicted forced expiratory volume (FEV1/pre), forced vital capacity (FVC), fraction of exhaled nitric oxide (FeNO; <25 ppb, normal; 25–50 ppb, moderate; >50 ppb, severe), total serum IgE, blood routine, and number of acute exacerbations.

### 2.3 Blood sampling and laboratory tests

Three (or two) tubes of venous blood, each 2 mL per tube, were drawn early in the morning on an empty stomach before treatment, 4 and 16 weeks after treatment, respectively. Eosinophil counts and percentages were examined using a fully automated blood analyzer (Mindray BC-2800, Shenzhen, China). Total IgE levels were measured by using electrochemiluminescence (Roche, Germany). This method could not distinguish between free IgE and IgE bound to omalizumab. sBT levels were measured using the ImmunoCAP trypsin immunoassay (Thermo Fisher Scientific, USA).

### 2.4 Pulmonary function measurement

Patients were required to rest for at least 15 min before the measurement and were evaluated using a pulmonary function testing machine (MasterScreen-PFT equipment, Jaeger Corp, Hoechberg, Germany). As instructed, patients pinched their noses, expelled air from their lungs to a functional residual volume, inhaled rapidly and forcefully, and then exhaled as quickly and forcefully as possible. The entire expiratory process was not less than 6 s, at least three measurements were required per patient. FEV1, FVC, and FEV1/pre were recorded.

### 2.5 Determination of exhaled FeNO

Nitric oxide was determined using a nitric oxide measuring instrument (Sievers Instruments, Boulder, USA). Patients, sitting with a nose clip, exhaled forcefully to reach the residual volume and then inhaled forcefully to achieve maximum lung capacity. The patients continued to exhale gradually at a fixed slow rate, and then the data were analyzed accordingly.

### 2.6 Treatment and observation

Based on the patient’s total serum IgE and body weight determined before treatment, the appropriate dosage of omalizumab and the frequency of administration (every 2 or 4 weeks) were determined using the dosage table. The dose of omalizumab was 75–600 mg per administration, which was injected subcutaneously at one site if the dose was ≤150 mg, or subcutaneously at one to four separate sites as needed if the dose was >150 mg. Injections were administered by trained physicians or nurses. According to the European Academy of Allergology and Clinical Immunology Guideline [21], omalizumab injections were closely observed for the occurrence of allergic reactions after the first three injections for 60 min, and the post-injection observation time was determined based on the individual patient’s response or history of previous adverse reactions.

### 2.7 Follow-up

Two weeks after the first treatment with omalizumab, patients were observed to confirm the suitability of omalizumab and to confirm that their asthma was in remission. The number of self-reported acute exacerbations, adverse effects, and adherence to medication, and medication use were recorded at follow-up visits during the 16-week treatment period (in-hospital visit during the fourth week, and by telephone or in-hospital visit every 4 weeks for the rest of the treatment period).

### 2.8 Assessment of efficacy

Asthma Control Test (ACT) score [22] and Pediatric Asthma Questionnaire Quality of Life Questionnaire (PAQLQ) [23] were utilized to assess the treatment efficacy. For children over 12 years of age, asthma was completely controlled (ACT = 25 points), basically controlled (ACT 20–24 points), and not controlled (below 20 points). Response to omalizumab treatment was defined by meeting any of the following criteria: (1) improvement in ACT score (post-treatment compared to pre-treatment) of ≥2 points in children 6–11 years of age; improvement in ACT score of ≥ 3 points in children 12 years of age and older, or ACT of ≤19 points pre-treatment, ≥20 points post-treatment. (2) Reduction in the frequency of acute asthma attacks by ≥50%. (3) Improvement of FEV1%pre ≥120 mL.

PAQLQ involves symptoms, activity, and emotional function with 23 questions, totaling a score of 161, where a higher score indicates a better quality of life. The efficacy evaluation was mainly based on the ACT score as a criterion.

### 2.9 Statistical analysis

Statistical analyses were performed using SPSS 20.0 software. The data’s normality was evaluated using the Shapiro–Wilk test. For normal distributions, measurements were displayed as mean value ± standard deviation (*X* ± *S*), and the Student’s t test was used for between-group comparisons; data with skewed distributions were expressed as medians (25th–75th percentiles interquartile range [IQR]), and the Mann–Whitney *U* test for between-group comparisons. Count data were expressed as frequencies (*n*) and ratios, and Chi-square or Fisher’s exact tests were used. The predictive value of the 4-week IgE/baseline IgE ratio and tryptase for efficacy in patients with moderate-to-severe asthma treated with omalizumab was assessed using receiver operating characteristic curve (ROC) and calculating the area under the ROC curve (AUC). Data were plotted using GraphPad Prism 8 (GraphPad, San Diego, CA) and the Hiplot (https://hiplot.org). *P* less than 0.05 was statistically significant.


**Ethical approval:** All procedures performed in this study involving human participants were in accordance with the ethical standards of the institutional and/or national research committee and with the 1964 Helsinki Declaration and its later amendments or comparable ethical standards. All subjects was approved by The Affiliated Suzhou Hospital of Nanjing University Medical School (No. 202203NUMS-15).

## 3 Results

### 3.1 Baseline characteristics of the children

**Figure 1:**
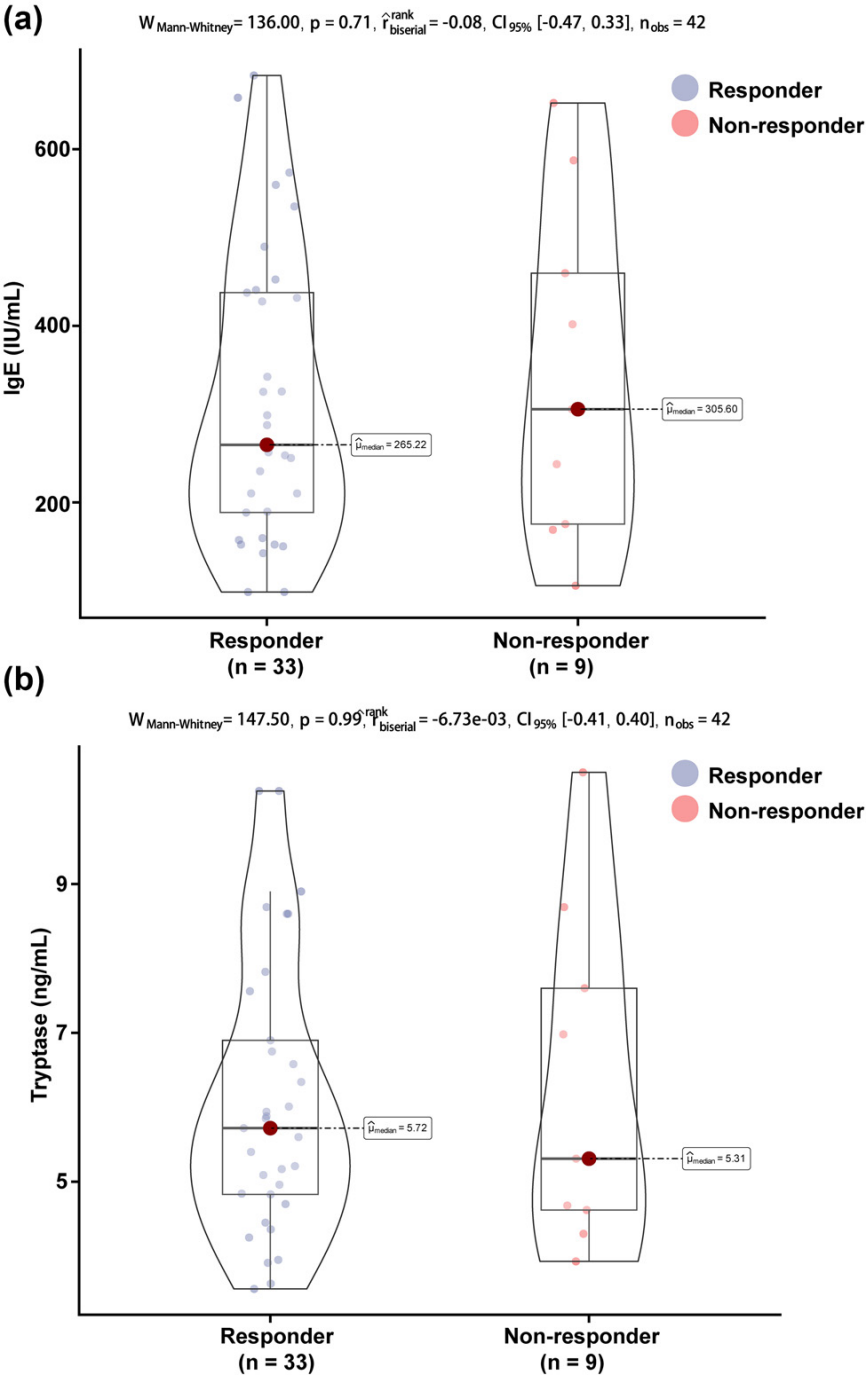
Serum total IgE and tryptase levels at baseline: (a) Serum total IgE and (b) tryptase levels.

Sixty-eight patients met the diagnostic criteria for the disease in this study, of whom 63 had completed 16 weeks of omalizumab treatment, six lacked IgE or tryptase results at 4 weeks of treatment, four had an interruption in follow-up due to noncooperation of the child’s guardian, and eight did not agree to sign the informed consent form. Forty-three children were enrolled and divided into a response group (*n* = 34) and a non-response group (*n* = 9) based on the efficacy of the children after 16 weeks of treatment. Table 1 shows the baseline clinical characteristics of these children. These children were between 6 and 14 years of age, 60.47% (26/43) were male, 74.42% (32/43) had moderate asthma, most of them had comorbid rhinitis (76.74%, 33/43), and 72% (31/43) had asthma onset in the first 16 weeks of treatment. No significant differences were observed in the clinical characteristics of the children in the two groups at baseline (all *P* > 0.05). In addition, baseline serum IgE levels (Figure 1A) were 265.22 (188.6, 437.56) IU/mL and 305.6 (175.52, 459.6) IU/mL in the response and non-response groups, respectively, with no statistically significant difference between the two groups (*P* = 0.71); for baseline tryptase (Figure 1B), the baseline levels in the response and non-response groups, respectively, were 10.77 (9.88, 12.61) ng/mL and 10.31 (9.67, 12.03) ng/mL, with no significant difference (*P* = 0.39).

**Table 1:** Comparison of baseline clinical characteristics between patients in the response and non-response groups

Variants	All (*n* = 43)	*R* (*n* = 34)	*N* (*n* = 9)	*P* value
Age (years)	8.7 (7.2, 12.7)	8.7 (7.2, 12.7)	8.7 (7.4, 12.8)	0.971
**Gender**				
Male	26 (60.47)	20 (58.82)	6 (66.67)	0.669
Female	17 (39.53)	14 (82.35)	3 (33.33)	
Weight (kg)	28.5 (23.6, 38.1)	28.5 (23.0, 38.1)	27.5 (25.2, 37.0)	0.895
Duration of asthma (month)	13 (10, 21)	13 (10, 21)	12.5 (9, 24)	0.881
**Degree of asthma**				
Moderate	32 (74.42)	25 (73.53)	7 (77.78)	0.795
Severe	11 (25.58)	9 (26.47)	2 (22.22)	
**Comorbid allergic diseases**				
Rhinitis	33 (76.74)	17 (70.83)	6 (66.67)	0.822
Conjunctivitis	8 (18.61)	5 (20.83)	3 (33.33)	
Nettle rash	2 (4.65)	2 (8.33)	0 (0.00)	
FEV1%pre	97.36 (90.85, 105.98)	97.36 (90.85, 105.98)	94.41 (92.97, 101.41)	0.935
FVC%	101.49 (97.31, 106.68)	101.05 (96.65, 106.69)	98.68 (94.19, 105.30)	0.08
FeNO	18.13 (13.36, 29.92)	18.13 (13.36, 29.92)	21.30 (15.20, 30.12)	0.758
Eosinophil count (×10^9^/L)	0.42 (0.34, 0.70)	0.42 (0.34, 0.70)	0.64 (0.35, 0.69)	0.676
Percentage of eosinophils	5.32 (4.22, 8.60)	5.32 (4.22, 8.60)	5.90 (4.60, 8.62)	0.969
Frequency of acute episodes before enrollment (episodes/16 weeks)	1 (0, 2)	1 (0, 2)	1 (0, 2)	1
ICS + LABA (μg/day)	366.60 (232.30, 849.75)	366.6 (232.3, 849.7)	542.3 (345.3, 825.3)	0.567
C-ACT	17 (16, 18)	17.0 (16.0, 18.0)	17.0 (15.0, 17.5)	0.323
ACT	16.5 (15.0 ,18.0)	16.5 (15.0, 18.0)	16.0 (14.2, 17.7)	0.62
PAQLQ	79.4 (72.5, 87.4)	79.4 (72.5, 87.5)	73.1 (71.3, 87.2)	0.611

Measurements are shown as (*X* ± *S*) or medians (IQR), and comparisons between groups were made using the Student’s t test or the Mann–Whitney *U* test; categorical data are shown as *n* (%) and compared using Mann–Whitney *U*. *P* < 0.05 was considered statistically significant. *R*, the group of clinical response patients; *N*, the group of non-response patients.

### 3.2 Clinical characteristics and adverse reactions of the treated children

As shown in Table 2, after 16 weeks of omalizumab treatment, eosinophil counts and percentages decreased (all *P* < 0.001), FeNO, ACT (including C-ACT) scores, and PAQLQ scores increased (all *P* < 0.001), and there were no significant changes in serum total IgE and pulmonary function indices (FEV1%pre and FVC%) (all *P* > 0.05). Notably, after 16 weeks of treatment, the acute asthma exacerbation rate was reduced, and the pre-treatment exacerbation rate of 72% was reduced to 23.26% (10/43), which was statistically significant (*P* < 0.001). Regarding the adverse effects of omalizumab, only a few children (four cases) had tolerable mild adverse effects, mainly manifested as redness and swelling at the injection site after the first injection in two cases, which subsided within 1 day; one case of mild headache, and one case of low-grade fever (which improved significantly after symptomatic treatment).

**Table 2:** Changes in clinical characteristics before and after 16 weeks of treatment

	Before treatment	After treatment	*P* value
FEV1%pre	97.36 (90.85, 105.98)	98.98 (93.82, 106.55)	0.334
FVC%	101.49 (97.31, 106.68)	102.49 (98.18,109.14)	0.378
FeNO	18.13 (13.36, 29.92)	10.13 (7.59, 17.01)	<0.001
Serum total IgE (IU/mL)	276.4 (178.8, 439.8)	288.5 (242.6, 520.3)	0.088
Eosinophil count (×10^9^/L)	0.42 (0.34, 0.70)	0.25 (0.16, 0.35)	<0.001
Percentage of eosinophils	5.32 (4.22, 8.60)	3.20 (2.23, 4.53)	<0.001
ICS + LABA (μg/day)	366.60 (232.30, 849.75)	169.66 (91.87, 325.06)	<0.001
C-ACT	17 (16, 18)	22.0 (20.5, 23)	<0.001
ACT	16.5 (15.0 ,18.0)	23.0 (21.0, 24.0)	<0.001
PAQLQ	79.4 (72.5, 87.4)	133.9 (126.1, 140.7)	<0.001

Measurements show medians (IQR) and were compared between groups using the Mann–Whitney *U* test; *P* < 0.05 was considered statistically significant.

### 3.3 Four-week IgE/baseline IgE ratio and tryptase levels after 4 weeks of treatment and the value of assessing efficacy

**Figure 2:**
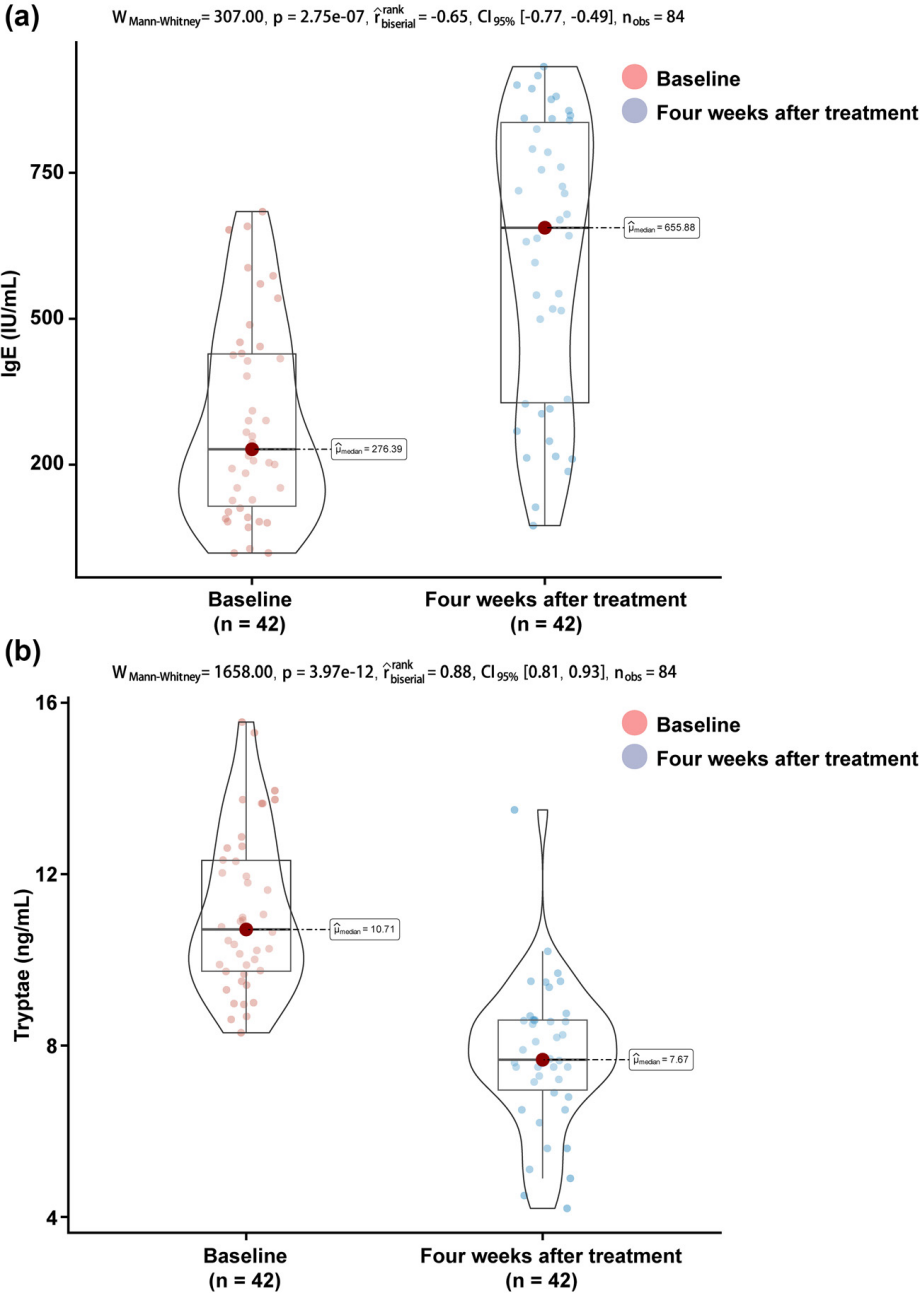
Comparison of serum total IgE and tryptase levels at baseline and after 4 weeks of treatment: (a) serum total IgE and (b) serum tryptase.

**Figure 3:**
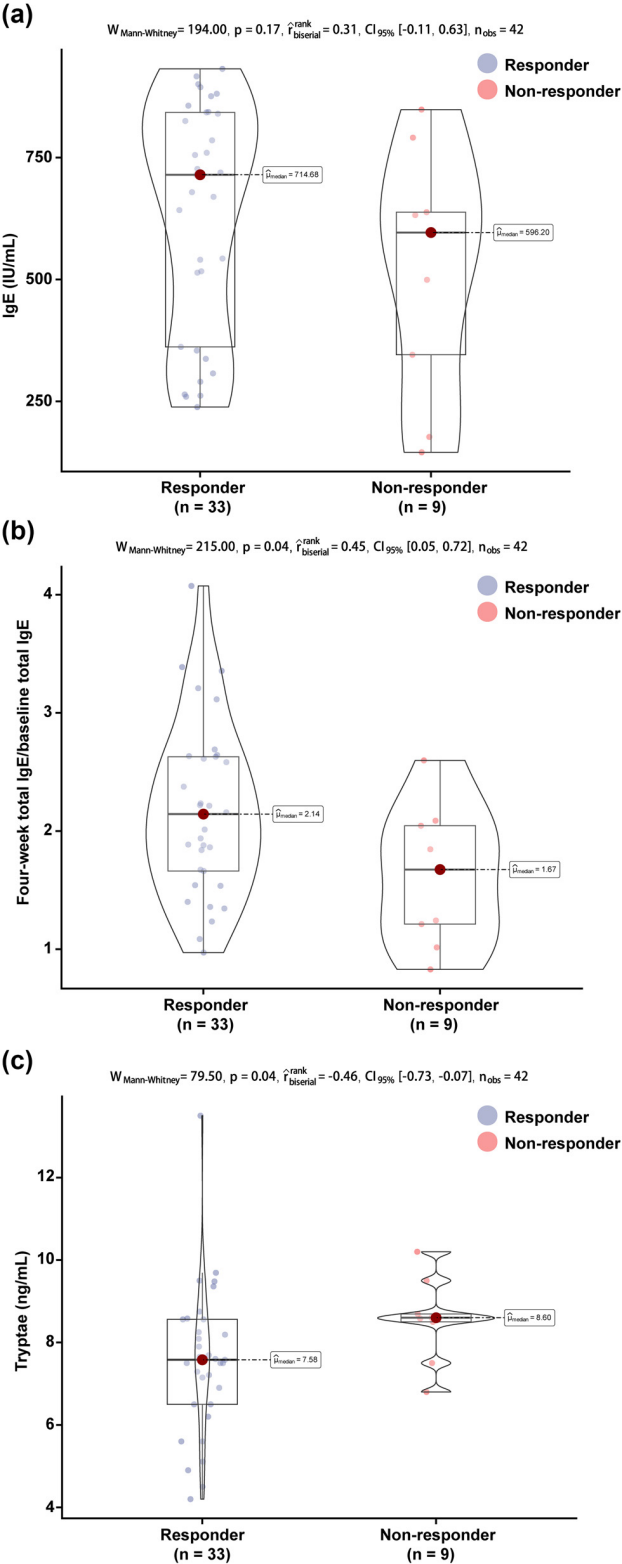
Comparison of serum total IgE, 4-week IgE/baseline IgE ratio, and tryptase levels after 4 weeks of treatment: (a) serum total IgE after 4 weeks of treatment; (b) four-week IgE/baseline IgE ratio after 4 weeks of treatment; and (c) tryptase after 4 weeks of treatment.

**Figure 4:**
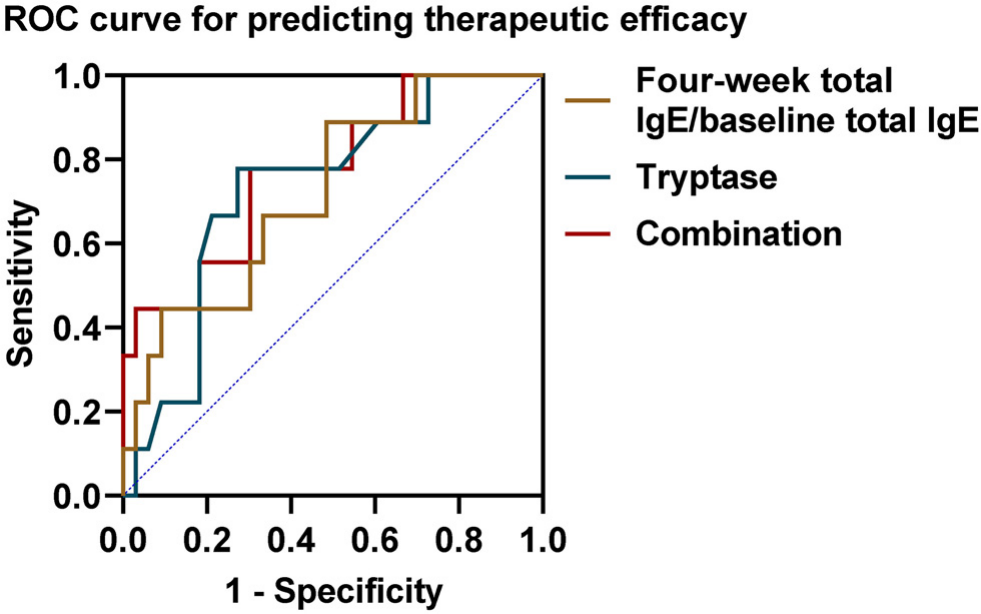
ROC curve and AUC to assess 4-week IgE/baseline IgE ratio, tryptase, and combined metrics in predicting patient outcomes.

Next we compared serum total IgE and tryptase levels between baseline and post-treatment in all children. The serum total IgE level increased substantially to 655.89 (356.09, 836.12) IU/mL after 4 weeks of treatment (Figure 2a, *P* < 0.001); and the serum tryptase was 5.31 (4.62, 7.60) ng/mL after 4 weeks of treatment, which was significantly higher than the level at baseline (Figure 2b, *P* < 0.001). Based on this study, 33 patients had a favorable response at the end of treatment. However, we did not observe a significant difference in total serum IgE between children in the response and non-response groups after 4 weeks of treatment (Figure 3a, *P* = 0.17); in particular, children in the response group had a higher 4-week IgE/baseline IgE ratio after 4 weeks of treatment than those in the non-response group (Figure 3b, *P* = 0.04), and lower tryptase than those in the non-response group (Figure 3c, *P* = 0.04). Neither the 4-week IgE/baseline IgE ratio nor the tryptase had a predictive effect on the efficacy of the children after 16 weeks of treatment with omalizumab, with AUC of 0.724 (95% CI 0.590–0.903, *P* = 0.060) and 0.732 (95% CI 0.579–0.907, *P* = 0.072). However, the combination of the two improved the predictive value and was statistically significant (AUC 0.774, 95% CI 0.679–0.920, *P* = 0.048) (Figure 4).

## 4 Discussion

Currently, the efficacy of omalizumab’s treatment of asthma is well established, but due to the heterogeneity of asthma, the universal guideline is to evaluate the treatment success after 16 weeks to decide on the continuation of omalizumab. The present study is a prospective follow-up study, and by analyzing the results of 43 patients with moderate-to-severe asthma after 4 and 16 weeks of treatment, we preliminarily demonstrated that the clinical efficacy of omalizumab for moderate-to-severe asthma may be able to be assessed based on the serum 4-week IgE/baseline IgE ratio and tryptase, which may help in early (up to 16 weeks or even at 4 weeks) clinical decision-making about whether to continue omalizumab.

The reasons for the ineffectiveness of omalizumab in the treatment of asthma are not fully understood. There are no standardized criteria for assessing the efficacy of targeted therapies for asthma. However, it has been demonstrated that the physician’s overall assessment of omalizumab treatment is the most meaningful measure of response to omalizumab therapy. The assessment methodology relies on both subjective and objective evaluation criteria [24,25]. In contrast, general pretreatment baseline clinical characteristics are less reliable as predictive markers. To assess the therapeutic efficacy, several indicators such as C-ACT/ACT score, PAQLQ score, ICS + LABA dosage, lung function, and the frequency of acute asthma exacerbations were used in this study. In a multicenter randomized trial, children treated with omalizumab showed a significant improvement in ACT scores and a significant reduction in the frequency of acute exacerbations [26]. In a survey of children aged 6–11 years, a significant reduction in ICS and LABA dosage was found after omalizumab treatment, reflecting increased patient control of asthma [27]. In terms of lung function, omalizumab-treated children also showed significant improvements in FEV1, further supporting its effectiveness in asthma management [28]. In the present study, most of the results were similar to those described above, and the children’s ACT/C-ACT scores improved after 1 week of omalizumab treatment, their quality-of-life scores improved, the use of inhaled ICS and LABA was reduced, and the frequency of acute asthma exacerbations was significantly reduced. However, there were no significant changes in serum total IgE and lung function indices (FEV1%pre and FVC%) between pre- and post-treatment. Another study found that although children on omalizumab also showed significant improvement in PAQLQ scores, no improvement in lung function was found [29]. The researchers concluded that long-term asthma may lead to permanent changes in airway structure, known as airway remodeling. This alteration may make recovery of lung function difficult and require longer observation.

The present study is the first to suggest that the 4-week IgE/baseline IgE ratio, in combination with tryptase after 4 weeks of treatment, predicts response in patients with moderate to severe asthma treated with omalizumab. In addition, this study reconfirmed that there is no significant correlation between serum total IgE levels and treatment efficacy at baseline and after 16 weeks of omalizumab treatment. A study reported that in eight patients with severe asthma, serum total, and free IgE treated with omalizumab were not the same in terms of long-term trends (104 weeks), but the majority peaked in serum total IgE after 4 weeks of treatment, and all patients had been on a decreasing trend in free serum IgE after treatment [30]. Our results also showed a substantial increase in serum total IgE levels after 4 weeks of omalizumab treatment compared with baseline level, although there was no difference between the response and non-response groups. In addition, although the IgE level after 4 weeks of treatment was not confirmed to be valuable in predicting omalizumab treatment in children with moderate-to-severe asthma, 4-week IgE/baseline IgE ratio was partially useful in predicting efficacy. At 4 weeks, the 4-week IgE/baseline IgE ratio increased, indicating an increase in the omalizumab-IgE complex. Omalizumab-IgE complex binds to and protects FcRn from lysosomal degradation, leading to an increase in total serum IgE. In addition, the complex itself can trap allergens to assist in the therapeutic effect of omalizumab [17].

Tryptase is involved in IgE-mediated inflammatory responses, and its elevated levels correlate with asthma severity or the degree of allergic reaction [31,32]. Its mechanisms of action in asthma include airway inflammation, airway reactivity, vascular remodeling, and modulation of immune response [20]. An important factor in asthma development is the Th2 type immune response. Th2 cells secrete cytokines such as IL-4, IL-5, and IL-13, which promote IgE production and eosinophil infiltration, thereby triggering asthma symptoms. Omalizumab promotes allergen desensitization by reversing the Th2 cell-like program and increasing the activity of allergen-specific Treg cells [33]. Tryptase has been reported to be involved in regulatory processes in Th2 cells [34]. In the present study, after 4 weeks of omalizumab treatment, tryptase levels were reduced in all children compared with baseline, and were lower in the response group. ROC analysis showed that the combination of 4-week IgE/baseline IgE ratio and tryptase had a predictive value in predicting patient outcomes after 16 weeks of treatment with omalizumab.

This study has some limitations. First, this study involved sample collection and follow-up, and the final sample size included was small, which may have some bias on the results. Therefore, additional multicenter and large-scale studies are needed in the future to further confirm the viewpoints of this study. Second, the follow-up time of this study was 16 weeks, based on the long-term nature of omalizumab treatment, it cannot be ruled out that the number of treatment-effective individuals will increase with the prolongation of omalizumab treatment. However, the ACT score after 16 weeks is reasonable as a determination of effectiveness in clinical practice and is more cost-effective than the determination of efficacy after prolonged anti-IgE treatment. Finally, the results of this study are applicable to the present study population and caution should be exercised in applying them to other diseases or to patients of different age groups.

## 5 Conclusion

The study suggests that 4-week IgE/baseline IgE ratio combined with tryptase levels may predict the efficacy of omalizumab anti-IgE therapy in asthmatic patients after 16 weeks, and that monitoring of serum total IgE and tryptase may predict the clinical efficacy of omalizumab.

## References

[1] Li LX, Lin SZ, Zhang RP, Chen SW. [Prevalence of pediatric asthma in the rural areas of China: a Meta analysis]. Zhongguo Dang Dai Er Ke Za Zhi. 2020;22(4):380–6. 10.7499/j.issn.1008-8830.1910164 PMC7389704 32312379

[2] Cano Garcinuno A, Mora Gandarillas I, Group SS. Early patterns of wheezing in asthmatic and nonasthmatic children. Eur Respir J. 2013;42(4):1020–8. 10.1183/09031936.00148712 23349448

[3] Simoneau T, Gaffin JM. Socioeconomic determinants of asthma health. Curr Opin Pediatr. 2023;35(3):337–43. 10.1097/MOP.0000000000001235 PMC10160003 36861771

[4] Yorgancioglu A, Reddel HK, Directors GBo, Committee GS. Global initiative for asthma: 30 years of promoting evidence-based asthma care. Allergy. 2023;78(7):1737–9. 10.1111/all.15714 36934290

[5] Lin JT, Wang WQ, Zhou X, Wang CZ, Huang M, Cai SX, , et al. [The level of asthma control in China from a national asthma control survey]. Zhonghua Jie He He Hu Xi Za Zhi. 2017;40(7):494–8. 10.3760/cma.j.issn.1001-0939.2017.07.002 28728272

[6] He Z, Armoni Domany K, Nava-Guerra L, Khoo MCK, Difrancesco M, Xu Y, et al. Phenotype of ventilatory control in children with moderate to severe persistent asthma and obstructive sleep apnea. Sleep. 2019;42(9):zsz130. 10.1093/sleep/zsz130 31175805

[7] Platts-Mills TAE, Schuyler AJ, Erwin EA, Commins SP, Woodfolk JA. IgE in the diagnosis and treatment of allergic disease. J Allergy Clin Immunol. 2016;137(6):1662–70. 10.1016/j.jaci.2016.04.010 PMC5406226 27264001

[8] Milger K, Schroeder I, Behr J, Meis T, Wulffen WV, Kneidinger N. Omalizumab rescue therapy for refractory status asthmaticus. Ann Intern Med. 2019;170(5):351–2. 10.7326/L18-0359 30458534

[9] Humbert M, Busse W, Hanania NA, Lowe PJ, Canvin J, Erpenbeck VJ, et al. Omalizumab in asthma: an update on recent developments. J Allergy Clin Immunol Pract. 2014;2(5):525–36e1. 10.1016/j.jaip.2014.03.010 25213045

[10] Kulus M, Hebert J, Garcia E, Fowler Taylor A, Fernandez Vidaurre C, Blogg M. Omalizumab in children with inadequately controlled severe allergic (IgE-mediated) asthma. Curr Med Res Opin. 2010;26(6):1285–93. 10.1185/03007991003771338 20377320

[11] Zhou H, Lu Y, Wu B, Che D. Cost-effectiveness of omalizumab for the treatment of inadequately controlled severe allergic asthma in Chinese children. J Asthma. 2020;57(1):87–94. 10.1080/02770903.2018.1544642 30507328

[12] Chinese Thoracic Society Asthma G. [Chinese expert consensus on the use of Omalizumab in allergic asthma (2021 version)]. Zhonghua Jie He He Hu Xi Za Zhi. 2022;45(4):341–54. 10.3760/cma.j.cn112147-20220115-00051 35381631

[13] Zhang M, Jin M, Zhou X, Lin J, Liu X, Liu C, et al. Effectiveness of omalizumab in patients with severe allergic asthma: A retrospective study in China. Respir Med. 2021;186:106522. 10.1016/j.rmed.2021.106522 34229289

[14] Korn S, Haasler I, Fliedner F, Becher G, Strohner P, Staatz A, et al. Monitoring free serum IgE in severe asthma patients treated with omalizumab. Respir Med. 2012;106(11):1494–500. 10.1016/j.rmed.2012.07.010 22884459

[15] Hsu CL, Shiung YY, Lin BL, Chang HY, Chang TW. Accumulated immune complexes of IgE and omalizumab trap allergens in an in vitro model. Int Immunopharmacol. 2010;10(4):533–9. 10.1016/j.intimp.2010.02.001 20139035

[16] Odajima H, Ebisawa M, Nagakura T, Fujisawa T, Akasawa A, Ito K, et al. Long-term safety, efficacy, pharmacokinetics and pharmacodynamics of omalizumab in children with severe uncontrolled asthma. Allergol Int. 2017;66(1):106–15. 10.1016/j.alit.2016.06.004 27507228

[17] Nishima S, Kozawa M, Milligan KL, Papadopoulos NG. Omalizumab and unmet needs in severe asthma and allergic comorbidities in Japanese children. Asia Pac Allergy. 2019;9(1):e7. 10.5415/apallergy.2019.9.e7 PMC6365659 30740355

[18] Esteves Caldeira L, Bernardino A, Paulino M, Costa C. Four-week total IgE/baseline total IgE ratio: Biomarker for omalizumab good response in chronic spontaneous urticaria real-life patients. J Allergy Clin Immunol Pract. 2023;11(12):3808–11. 10.1016/j.jaip.2023.09.010 37730088

[19] Atiakshin D, Buchwalow I, Samoilova V, Tiemann M. Tryptase as a polyfunctional component of mast cells. Histochem Cell Biol. 2018;149(5):461–77. 10.1007/s00418-018-1659-8 29532158

[20] Maun HR, Jackman JK, Choy DF, Loyet KM, Staton TL, Jia G, et al. An Allosteric anti-tryptase antibody for the treatment of mast cell-mediated severe asthma. Cell. 2019;179(2):417–31e19. 10.1016/j.cell.2019.09.009 PMC8403554 31585081

[21] Jensen-Jarolim E, Bachmann MF, Bonini S, Jacobsen L, Jutel M, Klimek L, et al. State-of-the-art in marketed adjuvants and formulations in allergen immunotherapy: a position paper of the European academy of allergy and clinical immunology (EAACI). Allergy. 2020;75(4):746–60. 10.1111/all.14134 31774179

[22] Koolen BB, Pijnenburg MW, Brackel HJ, Landstra AM, van den Berg NJ, Merkus PJ, et al. Comparing Global Initiative for Asthma (GINA) criteria with the Childhood Asthma Control Test (C-ACT) and Asthma Control Test (ACT). Eur Respir J. 2011;38(3):561–6. 10.1183/09031936.00173710 21406508

[23] Chantadul V, Poachanukoon O. Mini version of the pediatric asthma quality of life questionnaire (MiniPAQLQ): validity among Thai asthmatic children. J Med Assoc Thai. 2015;98(Suppl 2):S92–100. 26211110

[24] Li J, Kang J, Wang C, Yang J, Wang L, Kottakis I, et al. Omalizumab improves quality of life and asthma control in chinese patients with moderate to Severe Asthma: A randomized phase III study. Allergy Asthma Immunol Res. 2016;8(4):319–28. 10.4168/aair.2016.8.4.319 PMC4853509 27126725

[25] Oliveira MJ, Vieira M, Coutinho D, Ladeira I, Pascoal I, Ferreira J, et al. Severe asthma in obese patients: Improvement of lung function after treatment with omalizumab. Pulmonology. 2019;25(1):15–20. 10.1016/j.pulmoe.2018.01.005 30827349

[26] Busse WW, Morgan WJ, Gergen PJ, Mitchell HE, Gern JE, Liu AH, et al. Randomized trial of omalizumab (anti-IgE) for asthma in inner-city children. N Engl J Med. 2011;364(11):1005–15. 10.1056/NEJMoa1009705 PMC3093964 21410369

[27] Walker S, Burch J, McKenna C, Wright K, Griffin S, Woolacott N. Omalizumab for the treatment of severe persistent allergic asthma in children aged 6-11 years. Health Technol Assess. 2011;15(Suppl 1):13–21. 10.3310/hta15suppl1/02 21609649

[28] Palgan K, Zbikowska-Gotz M, Lis K, Chrzaniecka E, Bartuzi Z. Omalizumab improves forced expiratory volume in 1 second in patients with severe asthma. Postepy Dermatol Alergol. 2018;35(5):495–7. 10.5114/ada.2018.77241 PMC6232554 30429708

[29] Giubergia V, Ramirez Farias MJ, Perez V, Crespi N, Castanos C. Clinical impact of omalizumab treatment in children with severe asthma: Report of a local experience. Arch Argent Pediatr. 2019;117(2):e115–20. 10.5546/aap.2019.eng.e115 30869489

[30] Gon Y, Ito R, Maruoka S, Mizumura K, Kozu Y, Hiranuma H, et al. Long-term course of serum total and free IgE levels in severe asthma patients treated with omalizumab. Allergol Int. 2018;67(2):283–5. 10.1016/j.alit.2017.08.003 28927962

[31] Aniceto V, Dias MM, Melo JML, Trevisan-Neto O, Aragon DC, Maia LSM, et al. Serum baseline tryptase level as a marker for the severity of anaphylaxis. Int Arch Allergy Immunol. 2019;179(3):201–8. 10.1159/000497235 30893687

[32] Scarpelli MP, Keller S, Tran L, Palmiere C. Postmortem serum levels of IgE and mast cell tryptase in fatal asthma. Forensic Sci Int. 2016;269:113–8. 10.1016/j.forsciint.2016.11.001 27888720

[33] Abdel-Gadir A, Schneider L, Casini A, Charbonnier LM, Little SV, Harrington T, et al. Oral immunotherapy with omalizumab reverses the Th2 cell-like programme of regulatory T cells and restores their function. Clin Exp Allergy. 2018;48(7):825–36. 10.1111/cea.13161 PMC6021220 29700872

[34] Fu Z, Akula S, Thorpe M, Hellman L. Highly selective cleavage of TH2-promoting cytokines by the human and the mouse mast cell tryptases, indicating a potent negative feedback loop on TH2 immunity. Int J Mol Sci. 2019;20(20):5147. 10.3390/ijms20205147 PMC6834136 31627390

